# The Role of UID for the Usage of Verb Phrase Ellipsis: Psycholinguistic Evidence From Length and Context Effects

**DOI:** 10.3389/fpsyg.2021.661087

**Published:** 2021-05-26

**Authors:** Lisa Schäfer, Robin Lemke, Heiner Drenhaus, Ingo Reich

**Affiliations:** ^1^Collaborative Research Center 1102, Saarland University, Saarbrücken, Germany; ^2^Department of Modern German Linguistics, Saarland University, Saarbrücken, Germany; ^3^Department of Language Science and Technology, Saarland University, Saarbrücken, Germany

**Keywords:** ellipsis, VP ellipsis, information theory, uniform information density, rating study, self-paced reading study

## Abstract

We investigate the underexplored question of when speakers make use of the omission phenomenon verb phrase ellipsis (VPE) in English given that the full form is also available to them. We base the interpretation of our results on the well-established information-theoretic Uniform Information Density (UID) hypothesis: Speakers tend to distribute processing effort uniformly across utterances and avoid regions of low information by omitting redundant material through, e.g., VPE. We investigate the length of the omittable VP and its predictability in context as sources of redundancy which lead to larger or deeper regions of low information and an increased pressure to use ellipsis. We use both naturalness rating and self-paced reading studies in order to link naturalness patterns to potential processing difficulties. For the length effects our rating and reading results support a UID account. Surprisingly, we do not find an effect of the context on the naturalness and the processing of VPE. We suggest that our manipulation might have been too weak or not effective to evidence such an effect.

## 1. Introduction

When speakers want to get a message across, they often have the choice between ellipsis and the corresponding full form (1) and it is not always obvious which form to use. The underexplored question of why speakers sometimes prefer the ellipsis over the full form and sometimes do not is the topic of this paper, which we explore at the example of VP ellipsis.

VP ellipsis (Sag, 1976; Williams, 1977) is one of the most extensively studied omission phenomena in linguistics. The term refers to a kind of constituent ellipsis where the omitted element, i.e., the target of ellipsis, is a complete verb phrase. Only a corresponding auxiliary is left in the position of the omitted verb phrase (1).

(1) Sam played football
  - and Dean played football too.
  - and Dean did 〈play football〉 too.
  - and Dean should 〈play football〉 too.

The extensive literature on this phenomenon has focused on systemic questions like the modeling of the ellipsis site, the relation between the ellipsis site and its antecedent (or postcedent) and the licensing conditions of VP ellipsis (see e.g., Merchant, 2018; Reich, 2018, for recent overviews). Analogously, the psycholinguistic literature mainly addressed procedural aspects of the relation between antecedent and target such as complexity effects (see e.g., Frazier et al., 2000; Frazier and Clifton, 2001; Apel et al., 2007; Martin and McElree, 2008; Paape et al., 2017). However, to the best of our knowledge, the question of when and why speakers actually make use of VP ellipsis given that the corresponding full form is also available to them has not yet been investigated in the literature.

We pursue the hypothesis that VP ellipsis is preferred more strongly the more redundant the omitted material is, because this makes the most efficient use of the hearer's processing resources^1^. We base our account on the well-established information-theoretic Uniform Information Density (UID) hypothesis (Levy and Jaeger, 2007). According to UID, speakers tend to distribute information uniformly across utterances avoiding information minima caused by redundant material. We focus on two sources of redundancy that could impact the preference for VP ellipsis: the length of the redundant VP which leads to a longer redundant region and its predictability in context which causes a deeper redundant region. To test the predictions of UID with respect to length and predictability in context we first manipulate either the length of the redundant VP or its predictability in context and determine the naturalness of VP ellipsis in comparison to the corresponding full form. Second, we focus on the full forms and use a self-paced reading experiment to measure the processing effort associated with the redundant VP. This allows us to correlate differences in naturalness with potential processing difficulties caused by information minima.

This paper is structured as follows: In section 2, we present our information-theoretic account to the usage of VP ellipsis based on UID and discuss its predictions with respect to length and context effects. In section 3, we discuss length effects and present a naturalness rating study and a self-paced reading study on length effects. Section 4 is dedicated to effects of predictability in context and presents a pre-test, a rating study and a self-paced reading experiment. Section 5 summarizes our central findings and contributions.

## 2. Information-Theoretic Account to VP Ellipsis

The Uniform Information Density (UID) hypothesis (Levy and Jaeger, 2007) has been successfully applied to account for a variety of omission phenomena from acoustic reduction (Aylett and Turk, 2004; see Jaeger and Buz, 2017 for an overview), to the omission of functional elements such as relativizers (Levy and Jaeger, 2007), complementizers (Jaeger, 2010) and discourse markers (Asr and Demberg, 2015) in English, case markers in Japanese (Kurumada and Jaeger, 2015) and articles in German newspaper articles (Lemke et al., 2017), to the omission of content words, for instance the deletion of parts of the utterance in German fragments (Lemke et al., 2020) and the omission of preverbal subjects in Russian (Kravtchenko, 2014). In a recent study, Lemke et al.^2^ found that UID also constrains other elliptical phenomena such as sluicing. This makes UID a promising approach for describing the omission process of VP ellipsis where the ellipsis targets a whole VP with both function and content words.

**Figure 1:**
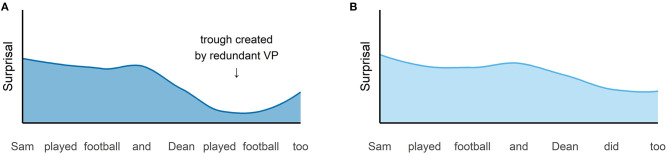
Hypothetical information density profiles for example (1): The surprisal values for the words of the full form **(A)** and for the words of the ellipsis **(B)** are plotted.

In the information theoretic framework, the *information* of an expression is defined as the negative binary logarithm of its conditional probability given context, i.e., −*log*_2_ *p*(*word*|*context*) (Shannon, 1948). Psycholinguistic research has established the synonymous term *surprisal* and has shown that information or surprisal indexes processing effort (Hale, 2001; Demberg and Keller, 2008; Levy, 2008). The central idea of the UID hypothesis is that communication is successful when surprisal or processing effort is distributed as uniformly as possible across an utterance. Such a uniform distribution avoids suprisal minima (*troughs*) and maxima above channel capacity (*peaks*) in the information density profile, i.e., it prevents that the processing capacities of the hearer are underutilized or exceeded. As a consequence, there are two ways in which an utterance can be optimized with respect to UID: First, speakers can omit predictable words which have low surprisal and would cause troughs in the information density profile. Second, speakers can smooth peaks by inserting a word before a very unpredictable word that is hard to process. If this insertion increases the predictability of the word that is hard to process, this reduces the processing effort on this word. With respect to VP ellipsis, the important point is the fact that surprisal minima are caused by redundant material. In full forms like (1-a), the repeated VP *played football* is redundant and we would in principle expect that a repetition of redundant material causes a surprisal minimum in the information density profile. In contrast, the ellipsis in (1-b) avoids such a minimum and thus smooths the information density profile. This results in a more uniform distribution and a more efficient use of the hearer's processing resources. This idea is illustrated in Figure 1^3^ using hypothetical surprisal values for example(1).

**Figure 2:**
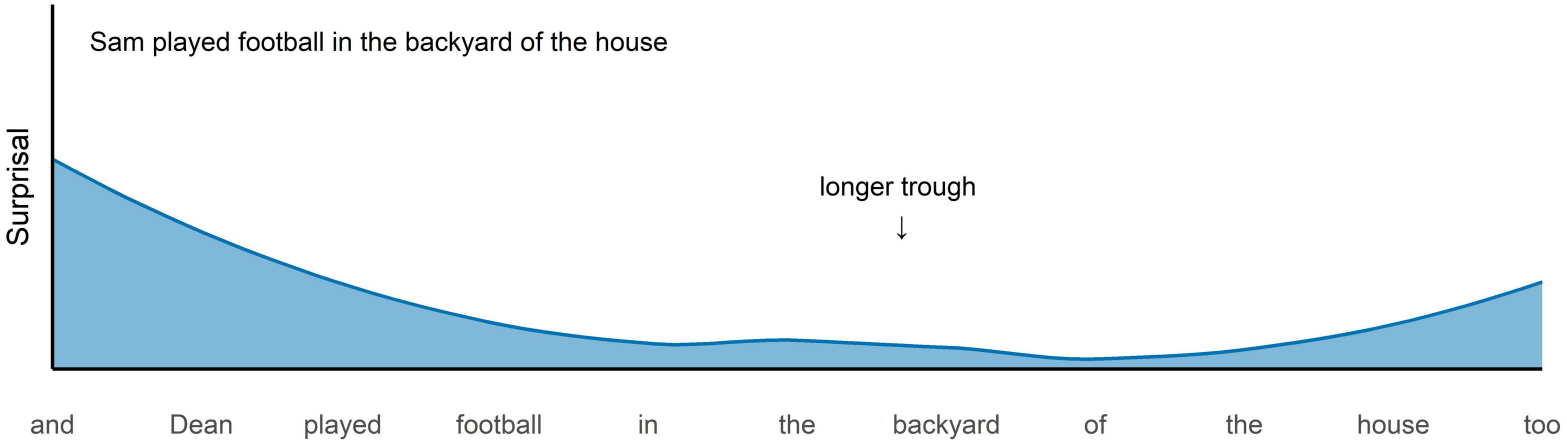
Hypothetical information density profile for the second conjunct of a longer version of example (1).

We investigate two potential sources of redundancy: the length of a VP and its predictability in context. Firstly, following UID we expect that the redundancy of a VP increases as a function of its length: Longer repeated VPs create longer regions of low information in the information density profile as shown in Figure 2. In this example the repeated VP is longer and hence causes a longer trough in the information density profile. Such longer regions make the utterance less efficient and we expect the pressure put on the speaker to omit the redundant part and to use VP ellipsis to be stronger in this case.

**Figure 3:**
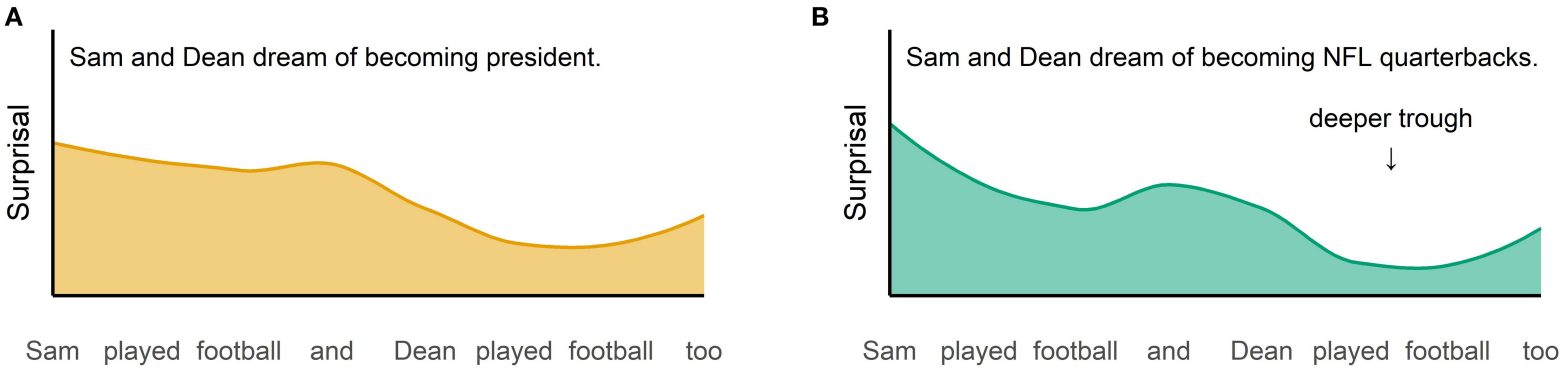
Hypothetical information density for profiles for example (1) in a neutral context like (2-b) **(A)** and a predictive context like (2-a) **(B)**.

Secondly, in line with UID also the predictability of the VP in context should impact its redundancy. Hence, exactly the same VP should create a deeper trough in the information density profile when it occurs in a predictive context compared to a neutral context. When the example in (1) is uttered in a predictive context like (2-a) compared to a neutral context like (2-b), the repeated VP *played football* becomes even more redundant because the context makes Dean more likely to play football (Figure 3). It thus conveys fewer information in this case and leads to a deeper trough in the information density profile. And such a deeper trough is equivalent to a less efficient use of the hearer's processing capacities. To avoid this, a speakers should have a stronger preference to use VP ellipsis in such predictive contexts.


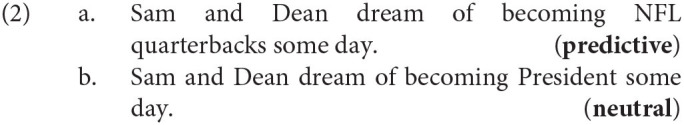


UID explains the production of utterances from the perspective of a speaker who performs audience design (Bell, 1984): She or he adapts her or his utterances as to facilitate comprehension for the hearer. We can assess the success of this audience design with naturalness rating and self-paced reading experiments which allows us to link the relative naturalness of ellipsis to the processing effort associated with the competing full forms.

Note that the UID predictions of avoiding redundancy are partially shared by accounts from research on anaphora^4^. First, Williams (1997, p. 603) postulates the principle *Don't Overlook Anaphoric Possibilities* (*DOAP*), according to which any opportunity to anaphorize text must be seized and a repeated phrase must be destressed (Williams, 1997, p. 595). Since Williams (1997) interprets deleted material as an instance of anphora, DOAP should also apply to VP ellipsis. Whenever deletion as extreme form of destressing is possible, speakers should make use of it and hearers should expect it. Realizing redundant material can in turn lead hearers to assume that there is a reason for this explicitness, e.g., in the form of a contrast. Consequently, if no such reason exists, hearers should reject the more redundant forms. A possible account based on the DOAP principle would hence predict that the repetition of redundant material is penalized, i.e., that it leads to degraded ratings. Conversely, the use of reduced forms such as VP ellipsis should be beneficial in that case and lead to better ratings.

Second, previous research has evidenced the so called *repeated-name penalty* (Gordon et al., 1993; Gordon and Hendrick, 1998; Almor, 1999) and the similar *overt pronoun penalty* in languages with null pronouns (Almor et al., 2017; Shoji et al., 2017): Participants read sentences more slowly when they contain a repeated name instead of a pronoun or an overt pronoun instead of a null pronoun. Gordon and Hendrick (1998, p. 390) argue that pronouns are primarily used to establish coreference, while names introduce entities into the discourse. Hence, coreference with names instead of pronouns requires additional processing effort resulting in increased reading times. Kertz (2010) adapts the concept of repetition penalties to VP ellipsis and rating data (see also Kim et al., 2011). She observes degraded ratings in contexts where a matched repeated VP was introduced by a parallel connective, calling this a *repeated verb phrase penalty*. A potential account based on the repetition penalties would consequently predict that processing difficulties caused by redundant material result in degraded acceptability.

The predictions of a possible DOAP approach and a potential repetition penalties account are partially consistent with those of the information-theoretic UID hypothesis: DOAP and the repetition penalties both predict degraded ratings through redundant material, which the latter account explains with processing difficulties. UID, however, explicitly makes gradual predictions: According to UID, a repeated VP is expected to be worse or more difficult to process, the longer it is or the more predictable it is in context. Possible accounts based on DOAP and the penalties would predict that any repetition of redundant material should be degraded and would not straightforwardly account for gradual or categorical effects of length or predictability. Hence, these predictions allow us to distinguish our UID account from the potential DOAP and repetition penalty accounts.

## 3. Length Effects

As outlined above, we expect, following UID, that the length of redundant material impacts the preference of a speaker to omit this material. More specifically, a longer redundant repeated verb phrase should be more likely to be omitted than a corresponding short repeated redundant verb phrase. We test this hypothesis first with a naturalness rating study which investigates the perception of long and short redundant verb phrases compared to their elliptical counterparts. This tells us whether the usage of ellipsis is motivated by a form of audience design: When VP ellipsis is preferred over full forms by hearers, speakers in turn should be more likely to use them to increase the efficiency of communication. Assessing whether repeated redundant verb phrases indeed lead to less efficient communication is the goal of the self-paced reading study on only the full forms. With respect to length we test whether the information minimum caused by redundancy is more severe when the repeated part is longer.

### 3.1. Experiment 1 – Naturalness Rating Study

In a 2 × 2 (Length: short vs. long × Form: full form vs. VPE) naturalness rating study we test the prediction that a long redundant verb phrase is more dispreferred than a short redundant verb phrase compared to the corresponding VP ellipsis.

#### 3.1.1. Materials

We constructed 32 items^5^ like (3) which consist in two coordinated main clauses with SVO word order respectively. The basic verb phrase is always a verb object pair like *play football* with the object being a DP without an overt determiner like *football*. We varied the Length of this verb phrase between short and long. In the short conditions we presented only the basic verb phrase, in the long conditions we expanded the verb phrase by a complex locative adverbial consisting of two nested prepositional phrases that defines more closely where the event described by the verb is happening. The verb phrase in the second conjunct was varied in its Form between the full form and VP ellipsis.


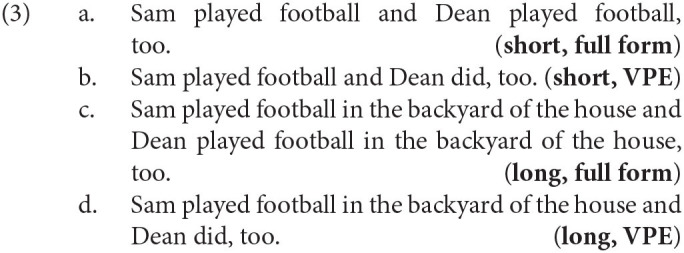


We mixed the items with 72 fillers, among which were 24 gapping constructions (4) and 24 constructions with a subject lacking (5), half of which were elliptical, half syntactically complete. We included these ellipses to ensure that our items did not stand out as being the only syntactically incomplete utterances and balanced ellipses and full forms across the experiment. Sixteen of the fillers were followed by polar comprehension questions that served as attention checks.

(4) Mary hates broccoli and John (hates) cauliflower.
(5) Cass entered the theatre after the start of the movie and (he) looked for his seat but it was already taken.

#### 3.1.2. Procedure

We recruited 48 self-reported native speakers of British English from the crowdsourcing platform Prolific Academic who received a compensation of £2. The survey was conducted over the Internet using the LimeSurvey survey presentation software^6^. Subjects were asked to rate the naturalness of the stimuli on a 7-point Likert scale where 1 was *completely unnatural* and 7 *completely natural*. Materials were distributed across four lists with a 2 × 2 Latin square design. Each subject saw each token set once and only in one condition. The 32 items were mixed with the 72 fillers and presented in pseudo-randomized order.

#### 3.1.3. Results

**Figure 4:**
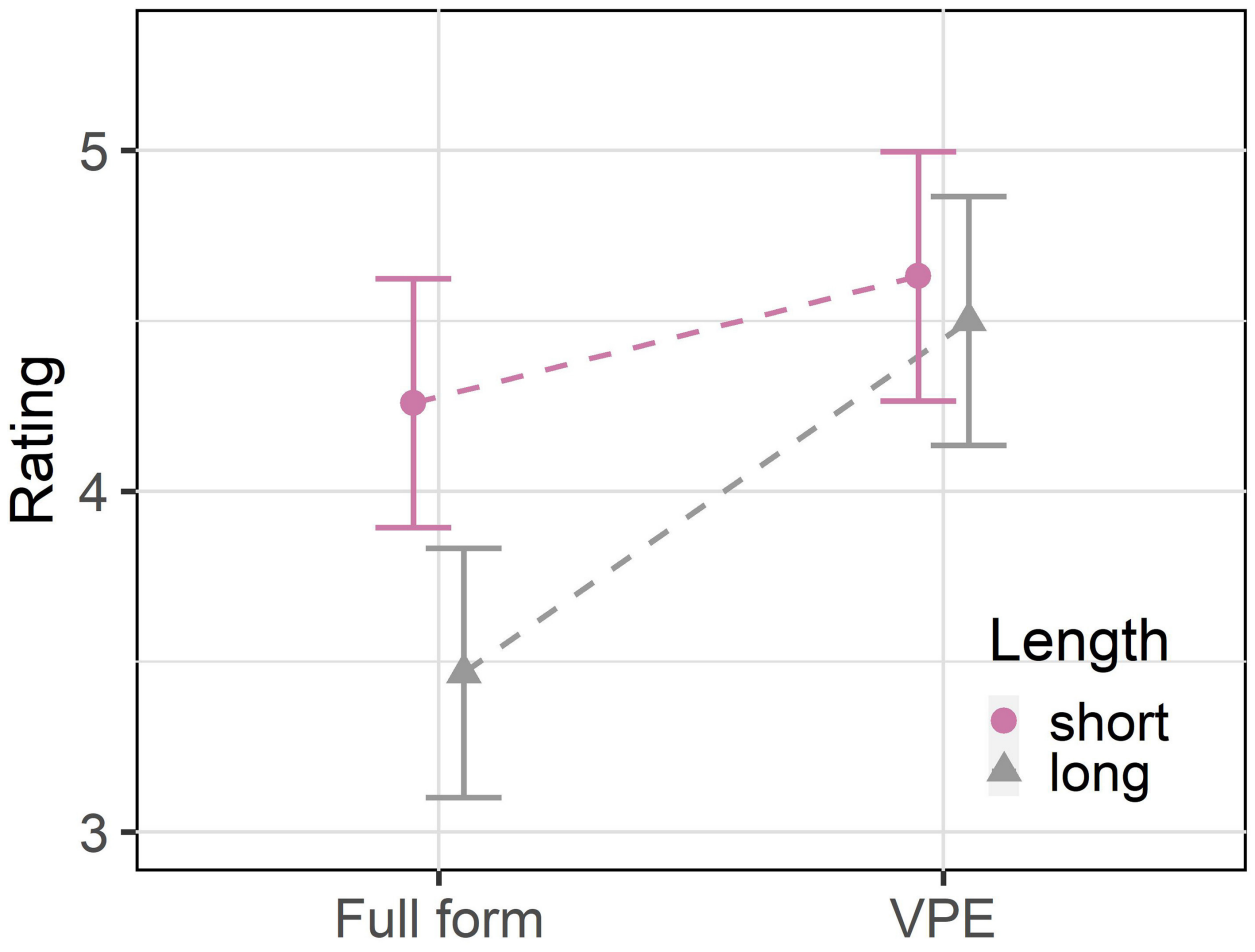
Mean ratings and 95% confidence intervals per conditions for experiment 1.

Before the main analysis we excluded 7 participants who failed our attention checks by answering more than the beforehand set threshold of 4 comprehension questions incorrectly. This threshold was established because at this point there is no significant difference to a purely random answering as evidenced by a chi-square goodness of fit test. We analyzed the remaining data in R (R Core Team, 2020) with cumulative link mixed models for ordinal data (Christensen, 2019). In all analyses in this paper we used a backward model selection procedure to find the final model: By performing likelihood ratio tests with the anova function we compared a model with and without an effect in question and continued with the simpler model if this did not significantly improve model fit. In our full model^7^ we model the ratings as a function of the two binary predictors Length and Form, the scaled and centered Position of the item in the experiment and all two way interactions between them. We used deviation coding for the two categorical variables with −0.5 and 0.5 as levels. We included the full random effects structure justified by the data (Barr et al., 2013), i.e., random intercepts for subjects and items and by-subject and by-item random slopes for Length, Form, Position and their two-way interactions. The final model (Table 1) contains a significant main effect of Length (χ^2^ = 29.45, *p* < 0.001) which shows that participants in general preferred utterances with short verb phrases over utterances with long verb phrases. The final model also revealed a significant main effect of Form (χ^2^ = 17.7, *p* < 0.001): The ratings for VP ellipsis were generally better than the ratings for the full forms. We found a significant interaction between Form and Length (χ^2^ = 11.85, *p* < 0.001) (see Figure 4): Full forms with a long repeated verb phrase are rated significantly worse than full forms with a short verb phrase as compared to utterances with VP ellipsis. A significant interaction between Form and Position (χ^2^ = 5.8, *p* < 0.05) and a significant main effect of Position (χ^2^ = 4.42, *p* < 0.05) show that in general the ratings became better in the course of the experiment and that they improved in particular for VP ellipsis which might indicate a familiarization effect.

**Table 1:** Fixed effects in the final clmm for experiment 1.

**Predictor**	**Estimate**	**SE**	**χ^2^**	***p*-value**	
Form	–1.24	0.27	17.7	< 0.001	^***^
Length	0.88	0.14	29.45	< 0.001	^***^
Position	0.28	0.13	4.42	< 0.05	^*^
Form:Length	1.1	0.3	11.85	< 0.001	^***^
Form:Position	–0.3	0.12	5.8	< 0.05	^*^

* *p < 0.05*,

^**^p < 0.01,

*** *p < 0.001*.

#### 3.1.4. Discussion

Our naturalness rating study confirms the prediction of the UID hypothesis on length effects: The results show that while participants overall prefer utterances with short repeated verb phrases and with VP ellipsis, long redundant full forms are particularly dispreferred as compared to the corresponding VP ellipsis conditions.^8^ This is in line with the prediction that from a hearer perspective VP ellipsis is particularly preferred in the long conditions where the full form would create a long surprisal minimum. If a speaker performs audience design, she or he should take the hearer perspective into account and there should be a stronger pressure to omit the redundant material.

The main effect of form that shows a general preference for VP ellipsis over full forms is also expected by UID: Participants favor the more reduced form of an ellipsis over the redundant repetition of identical material in the full form. The repeated verb phrase is redundant in both length conditions because it is completely identical to the verb phrase in the first conjunct. This means that even in the short conditions two words are used to communicate what in the ellipsis conditions can be said with a single *did*. Ellipsis hence avoids a trough in the ID profile that would be caused by the redundant repetition of the identical verb phrase. The result that redundant repetitions are generally dispreferred is also in line with the DOAP principle of Williams (1997) and with the repetition penalties (e.g., Gordon et al., 1993; Kertz, 2010), but these approaches cannot account for the observed interaction, i.e., they do not straightforwardly predict the gradual nature of the length effect.

Participants seem to generally prefer shorter utterances which might be related to the fact that the locative adverbials consisting of two PPs are more demanding than the very simple plain VPs. In sum, experiment 1 is in line with the UID predictions: Speakers prefer VP ellipsis especially when it avoids the redundant repetition of a long verb phrase.

### 3.2. Experiment 2 – Self-paced Reading Study

While experiment 1 showed the expected naturalness pattern, we need to complement it with an on-line self-paced-reading study to test the UID predictions about processing effort. According to our UID account the degraded ratings for the long redundant full forms are caused by an information minimum that underutilizes the hearer's processing capacities. To test this prediction we use a 1 × 2 (Length: short vs. long) self-paced reading paradigm. We measure the reading times for the redundant verb phrase to see whether participants indeed speed up on this region. Our UID account predicts that a redundant verb phrase is read relatively faster when it is longer than when it is shorter.

#### 3.2.1. Materials

We used only the full forms of the same 32 items and 72 fillers that were tested in experiment 1 including the 16 comprehension questions that served again as attention checks. We measured reading times on the first and the second verb phrase as illustrated in (6). The items were expanded by a spillover region always consisting in a clause introduced by *whereas* or *while* which described a different action performed by a third person. This prevents a wrap-up effect on the final word of the second verb phrase and makes the two verb phrases more comparable.


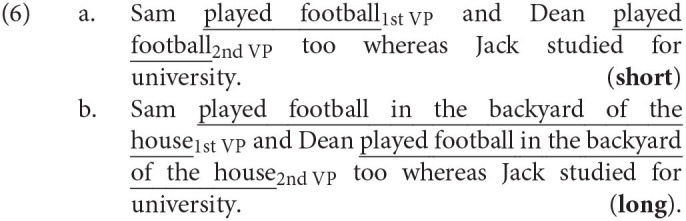


#### 3.2.2. Procedure

We recruited 96 self-reported native speakers of British English from the crowdsourcing platform Prolific Academic who were paid £2. None of the participants had taken part in experiment 1. The experiment was conducted over the Internet using IBEX^9^. Subjects read the stimuli in a centered self-paced reading paradigm. Materials were presented word by word on the screen. The experiment was preceded by a practice phase with 7 sentences and 2 comprehension questions to familiarize subjects with the procedure. Materials were distributed across two lists with a Latin square design. Each subject saw 32 items (16 per condition) which were mixed with the 72 fillers and presented in fully randomized order. Sixteen of the fillers were followed by attention checks in the form of polar comprehension questions.

#### 3.2.3. Pre-processing

The dependent variable that we use in our analysis are residualized cumulated reading times (RCRT in what follows) which we compare between the first and the second verb phrase. To obtain these reading times we first excluded all by-word reading times that were faster than 90 ms and slower than 3,000 ms. Since we compare the reading times of a whole region of interest, i.e., the whole verb phrase as underlined in (6), we excluded all regions that had become incomplete due to the by-word exclusions. These exclusions resulted in a loss of approximately 2% of the regions of interest. For each region of interest we summed up the plain by-word reading times. These cumulated reading times were then residualized based on the item data of all participants. That means that the cumulated reading times were normalized for length per participant by using the residuals of a linear model computed on the items of all participants with reading times as a function of number of characters (see Gibson and Levy, 2016).^10^ This allows us to compare the speed-up on the second verb phrase between short and long verb phrases despite the varying number of characters.

#### 3.2.4. Results

We excluded the data of 26 participants who had answered more than 4 of our 16 comprehension questions incorrectly.^11^ We analyzed the remaining data with linear mixed effects models (Bates et al., 2015) in R. Our full model contained the RCRT as dependent variable and the binary predictors Length (short vs. long VP) and VP (first vs. second VP), the scaled and centered Position of the trial in the experiment and all two-way interactions between the predictors. We coded the two categorical variables with −0.5 and 0.5 respectively using deviation coding. We included a random intercept for items, a by-item random slope for Length and a by-subject random slope for VP.^12^ Given that we use a dependent variable that is already normalized for subject and length effects and given that the two verb phrases are always identical for each item we used this informed random effects structure.

**Figure 5:**
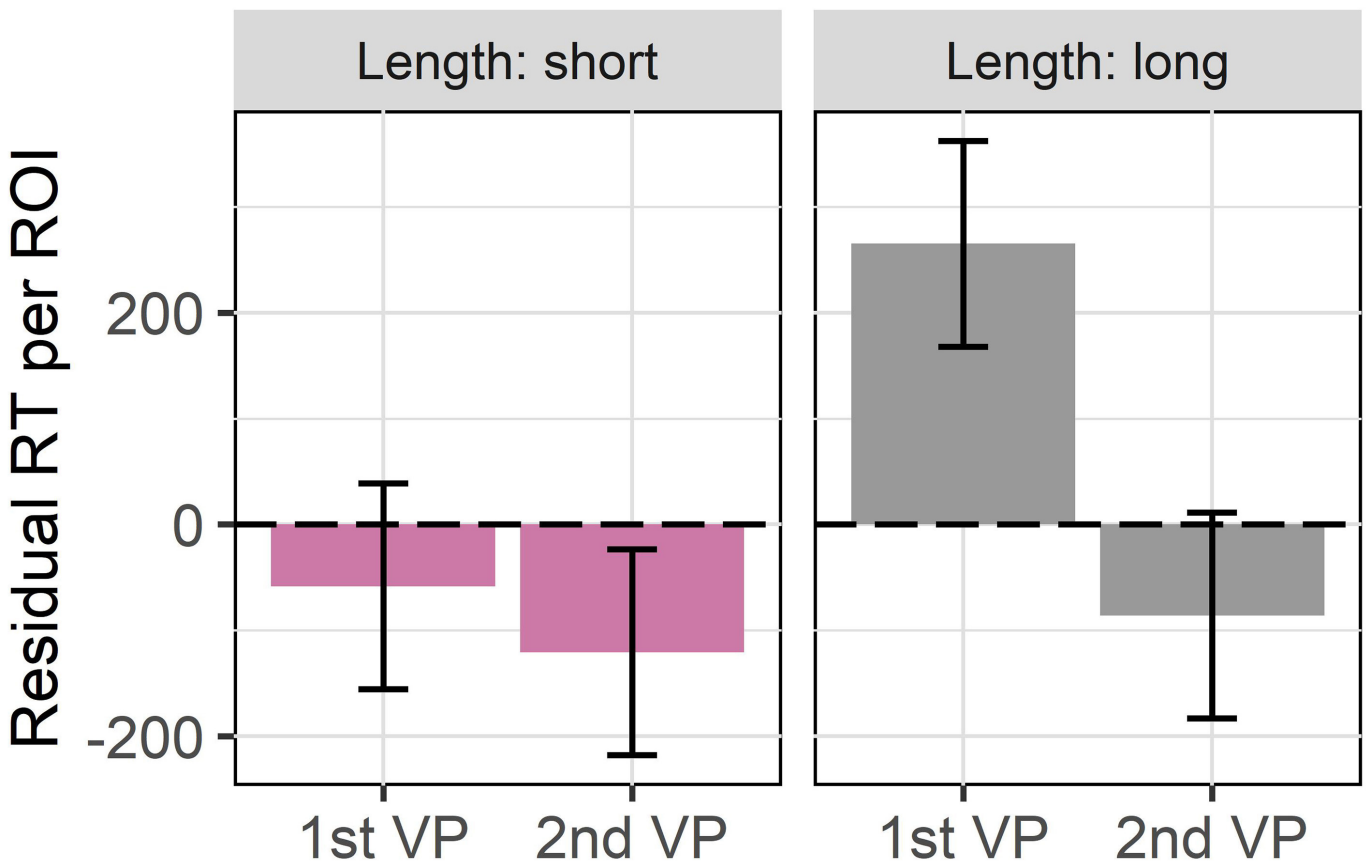
Mean residual cumulated reading times per region of interest and 95% confidence intervals for experiment 2.

The final model (Table 2) revealed a significant main effect of VP (χ^2^ = 39.22, *p* < 0.001): Participants read the second (redundant) verb phrase faster than the first (non-redundant) verb phrase. The model also revealed a significant main effect of Length (χ^2^ = 13.07, *p* < 0.001): Participants were overall faster on the short verb phrases. The model contained a significant interaction between Length and VP (χ^2^ = 95.82, *p* < 0.001) (see Figure 5): The speed-up on the second verb phrase as compared to the first was especially fast for the long verb phrases. Furthermore, the final model contained a significant main effect of Position (χ^2^ = 730.55, *p* < 0.001) and significant interactions of Position with Length (χ^2^ = 209.46, *p* < 0.001) and with VP (χ^2^ = 5.05, *p* < 0.05). Participants became notably faster during the experiment which indicates an increased familiarity with the task, in particular they speeded up on the first verb phrase and on the long verb phrases.

**Table 2:** Fixed effects in the final lmer for experiment 2.

**Predictor**	**Estimate**	**SE**	**df**	**χ^2^**	***p*-value**	
Length	–179.85	45.48	30.99	13.07	< 0.001	^***^
VP	206.38	28.61	68.60	39.22	< 0.001	^***^
Position	–211.09	7.48	4291.06	730.55	< 0.001	^***^
Length:VP	–289.88	29.46	4265.28	95.82	< 0.001	^***^
Length:Position	219.23	14.97	4287.63	209.46	< 0.001	^***^
VP:Position	–33.16	14.75	4275.01	5.05	< 0.05	^*^

* *p < 0.05*,

^**^p < 0.01,

*** *p < 0.001*.

#### 3.2.5. Discussion

The result of the self-paced reading study is in line with the UID prediction: The speed-up on the second verb phrase is bigger for the long conditions than for the short conditions. A long redundant verb phrase should thus create a longer region of low surprisal and result in a more severe underutilizing of the hearer's processing resources. This is exactly what is reflected in the degraded naturalness ratings for the long full form in the rating study in section 3.1. Hence, the reading study shows that the degraded ratings can be traced back to a non-optimal information density profile.

The reading study furthermore showed that participants were faster on the short verb phrases even after normalizing for the differing number of characters. This might be due to the fact that there is less material to be integrated when processing shorter utterances. Additionally there was a general speed-up between the first and the second verb phrase. Since participants already know the verb phrase when they encounter it for the second time, they may consequently read it faster. The massive position effects observed in the analysis indicate that participants became more and more familiar with the experimental design and the structures. It might be the case that the long redundant verb phrases are particularly marked and that participants are slow when they first encounter them, but become faster in the course of the experiment as a familiarization effect.

In total, the results of this reading study are in line with UID: They suggest that the degraded ratings from experiment 1 are indeed caused by a non-optimal information density profile with a long trough.

## 4. Context Effects

Experiments 1 and 2 showed that redundant structures are dispreferred and harder to process as predicted by UID. In what follows we explore a second source of redundancy that in contrast to length allows us to keep the target verb phrase constant across conditions: the predictability through context. The central idea is that a verb phrase is the more redundant, i.e., the less informative, the more predictable it is based on the previous linguistic context (2), repeated here as (7). For instance, in (8), Dean should be more likely to also play football if he wants to become a NFL quarterback (7-a) than if he wants to become President (7-b).


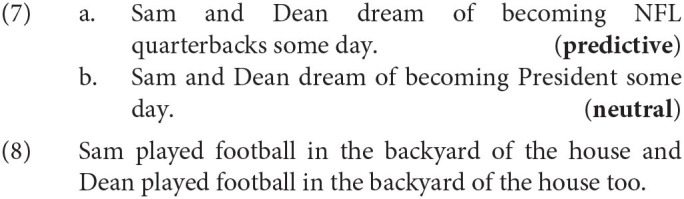


Just as with the length effects, we test this prediction with a naturalness rating experiment and a self-paced reading study. Again, we want to measure the naturalness of ellipsis as compared to the corresponding full forms and to trace back possible differences to processing as indexed by reading times. Before our actual experiments, we conducted a pre-test to test whether our contexts were indeed either predictive or neutral.

### 4.1. Experiment 3 – Pre-test

Up to now we have only assumed that the context (7-a) is more predictive than the context (7-b). We verify this assumption with a pre-test in which we obtain estimates for the likelihood of the second conjunct in context, independent of ellipsis. This pre-test should evidence that our verb phrases are likely in the predictive contexts and significantly less likely in the neutral contexts. Based on the results we select those items for the subsequent rating and reading study for which we find a significant difference in likelihood between the predictive and neutral context condition. Additionally, it is crucial to avoid that our neutral contexts are not only less predictive but implausible. Implausible contexts could be problematic for at least two reasons: First, if participants cannot make sense of the respective items, this might lead to an overall rejection of the neutral conditions. This would mask any fine-grained UID effects. Second, being confronted with too many implausible contexts could lead participants to abandon predictive processing during the rating study (see e.g., Fine et al., 2013; Brothers et al., 2017, who show that participants rapidly adapt their predictions during sentence comprehension) and this could override the predictability manipulation altogether. Therefore, we needed to assure that our neutral contexts make the critical verb phrases significantly more likely than implausible controls.

#### 4.1.1. Materials

We constructed a presumably predictive and a presumably neutral context sentence respectively for each of the 32 items from experiments 1 and 2 which were slightly adapted to better fit to the contexts. We tried to keep both context conditions as parallel as possible by either varying only the object of the VP or in some cases an embedded VP.^13^

Instead of presenting the coordinated structures to participants we used only the second conjunct, i.e., the one that will be targeted by VP ellipsis in the actual experiment (9). This way we ensured that we only test the predictability of the target verb phrase in the given context. In order to have more material on which we could measure reading times in the planned reading experiment, we used the long variants from experiments 1 and 2.


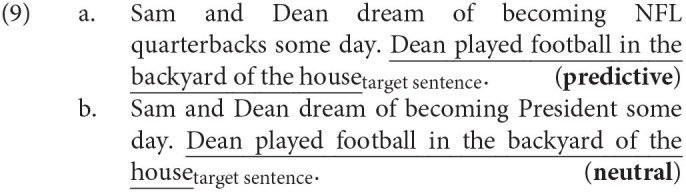


We mixed our items with 90 fillers including 32 similar items for another experiment with two context sentences and 34 script-based (Schank and Abelson, 1977) fillers with one context sentence. For half of these fillers the context made the target sentence predictable, for half not. The remaining 24 fillers were pre-tested stimuli, 12 with 1 context sentence, 12 with 2 context sentences, of which half were implausible because they contained severe script violations as exemplified in (10).^14^ We included them as controls to verify that our neutral contexts were not implausible, i.e., that the ratings for items with neutral contexts are significantly higher than the ratings for items with implausible contexts.


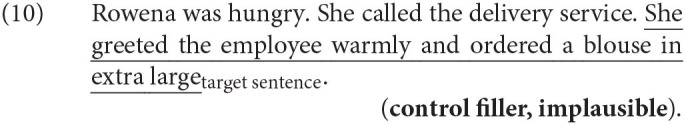


#### 4.1.2. Procedure

We recruited 48 self-reported native speakers of American English from Prolific Academic who had not participated in experiments 1 and 2 and compensated them with £2.50. They had to rate how likely it is that the event described by the target sentence, which was presented in bold face, happens in the given context using a slider scale from 0 (*cannot happen*) to 100 (*must happen*). The items were distributed across two lists with a Latin square design. Each subject rated 32 items (16 with a predictive, 16 with a neutral context) which were mixed with the fillers and presented in fully randomized order.

#### 4.1.3. Results

**Figure 6:**
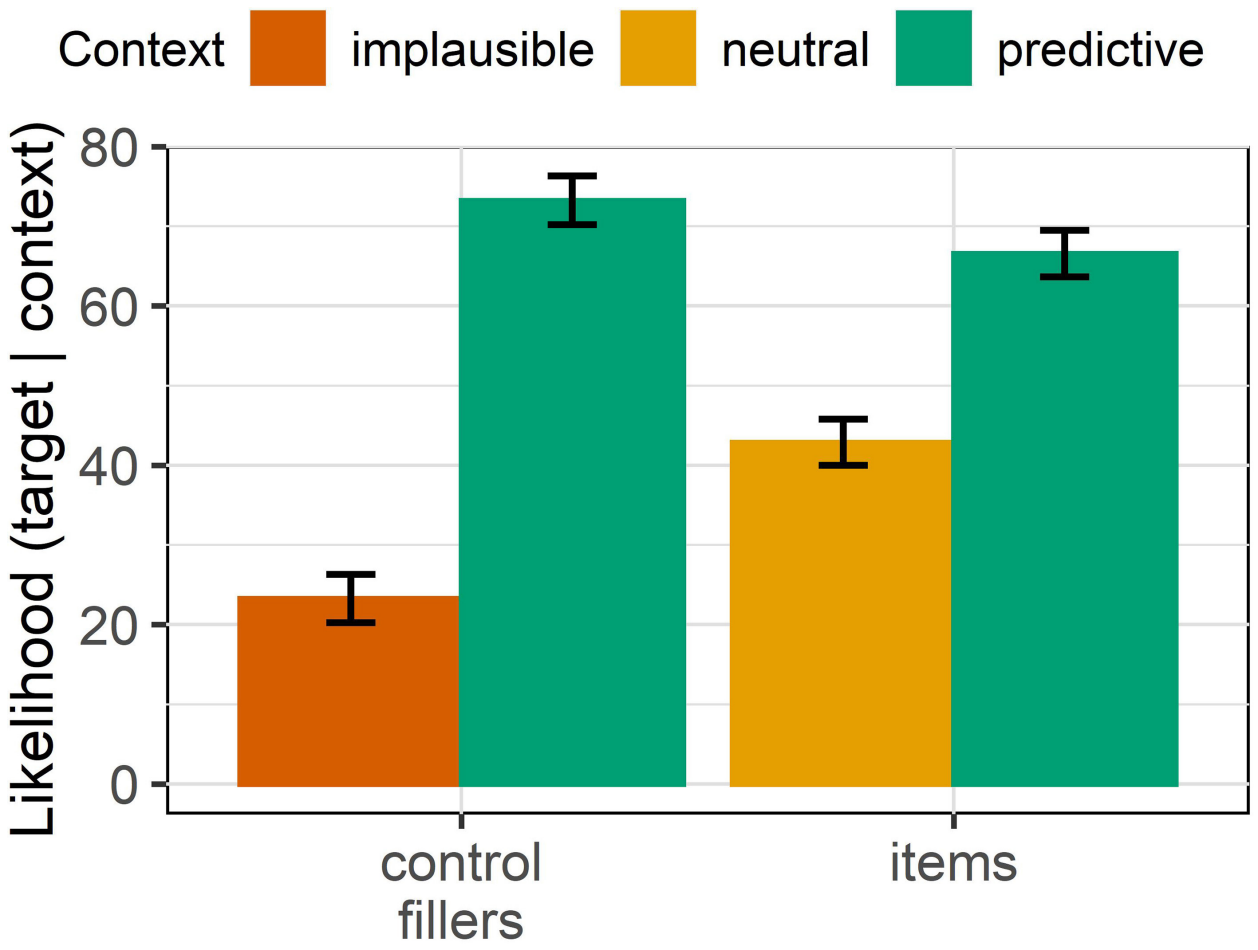
Mean likelihood ratings and 95% confidence intervals for items and control fillers in experiment 3. The implausible conditions of the control fillers were rated as significantly less likely than the neutral items.

Figure 6 shows the mean likelihood ratings and 95% confidence intervals for our items and the implausible (and corresponding predictive) controls. The implausible context fillers had a mean likelihood rating of 23.08 points (σ = 24.73) whereas the neutral context conditions of our items were rated with an average of 42.82 points (σ = 25.19) This indicates that our items are not implausible, but only less probable. This is confirmed by the results of a linear mixed effects model (Bates et al., 2015) on a subset of the data consisting of the control fillers and the items. For the analysis we collapsed the implausible and the neutral context conditions which are jointly contrasted with the predictive conditions. We model the likelihood score as a function of stimulus type and context and find a significant interaction between both predictors in the expected direction (χ^2^ = 29.58, *p* < 0.001): The implausible fillers received significantly lower likelihood ratings than the neutral items. This indicates that our neutral contexts should be plausible and we should receive valid ratings for them.

In order to select the items for the rating and the reading experiment, we assessed for each item whether the likelihood rating for the predictive context was significantly higher than for the neutral context. We compared the mean rating for the neutral context condition to the mean rating for the predictive context condition for each token set separately with one-sided Wilcoxon-tests in R. For 24 of 32 items the rating for the predictive context was significantly higher than for the neutral context, so we selected them for our main experiments.

### 4.2. Experiment 4 – Naturalness Rating Study

Our UID account predicts that a redundant verb phrase is more likely to be omitted. While experiment 1 and 2 showed that this redundancy increases as a function of the verb phrase's length, a second source of redundancy could be predictability in context. A repeated verb phrase should also be more redundant if it is likely given the previous context. We expect that this additional redundancy creates a deeper information minimum in the full forms which leads to degraded naturalness ratings. We test this with a 2 × 2 (Context: predictive vs. neutral × Form: full form vs. VPE) naturalness rating study.

#### 4.2.1. Materials

We used the 24 items which we had selected with the pre-test including predictive and neutral contexts. We reinserted the first conjunct to the target sentence (11) so that the target sentences were basically identical to the long conditions of experiments 1 and 2 and added a sentence-initial adverbial.


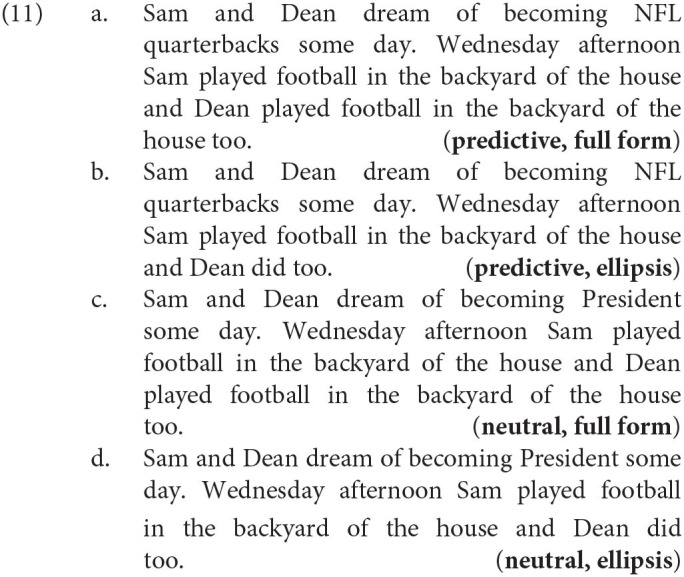


The items were mixed with 36 fillers which resembled the items in consisting of a context sentence and a target sentence with two coordinated verb phrases. Their purpose was to avoid a habituation effect caused by the structure of our items. Since the structure of our items was relatively constant, subjects could anticipate a redundant verb phrase as soon as they encounter a verb phrase followed by an *and*. This could overwrite or weaken the predictability manipulation of the verb phrase that we intended through the context sentence. Therefore we created 12 filler sentences where a completely different conjunct followed the coordination (12), 12 fillers where we changed the prepositional phrase but maintained the basic verb phrase (13) and 12 fillers where the prepositional phrase was kept constant but the verb phrase changed (14). For half of the sentences with a repeated phrase (*n* = 12) we substituted this phrase with an ellipsis (13) or a pro-form such as *there* in (14). This way, participants could not anticipate an identical second verb phrase when encountering *and*.


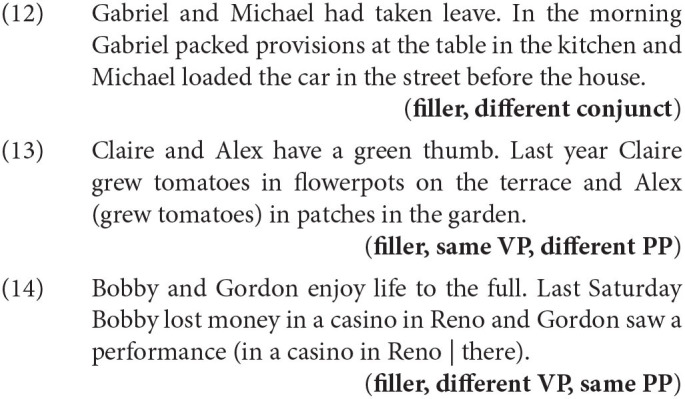


We further included 24 items from another experiment and 24 fillers which both had a structure similar to our items and each of which were half elliptical. This again was intended to ensure that our items did not stand out as the only syntactically incomplete utterances. Sixteen of the fillers were followed by polar comprehension questions asking either for information from the context or the target sentence that served as attention checks.

#### 4.2.2. Procedure

We recruited 96 self-reported native speakers of American English on Prolific Academic who had not taken part in any of the previous experiments. They were compensated with £2. We presented the survey over the Internet using IBEX. Subjects rated the naturalness of the critical utterance which was set in italics on a 7-point Likert scale (7 was *completely natural*). Materials were distributed across four lists with a Latin square design. Each subject saw each token set once and only in one condition. The Form of the items was varied between subjects, i.e., 48 subjects saw only ellipses, 48 subjects only full forms in order to avoid floor effects for the marked redundant full forms.

#### 4.2.3. Results

Before the analysis we excluded 13 subjects who had not passed our attention checks by answering more than 4 of 16 comprehension questions incorrectly. The threshold was set analogously to experiment 1 in section 3.1.3. The data of the remaining 83 subjects was analyzed using cumulative link mixed models (Christensen, 2019) in R following the procedure described for experiment 1 in section 3.1.3. The full model contained the ratings as an ordinal dependent variable and as independent variables the binary Form predictor, the numerical mean pre-test score by item and condition indicating Predictability, the scaled Position of the trial in the experiment and all two-way interactions between them. The categorical variable Form variable was transformed to −0.5 and 0.5 respectively using deviation coding. We included random intercepts for subjects and items and by-subject random slopes for Predictability and Position, as well as by-item random slopes for all three predictors and a by-item random slope for the interaction between Predictability and Form.^15^

**Figure 7:**
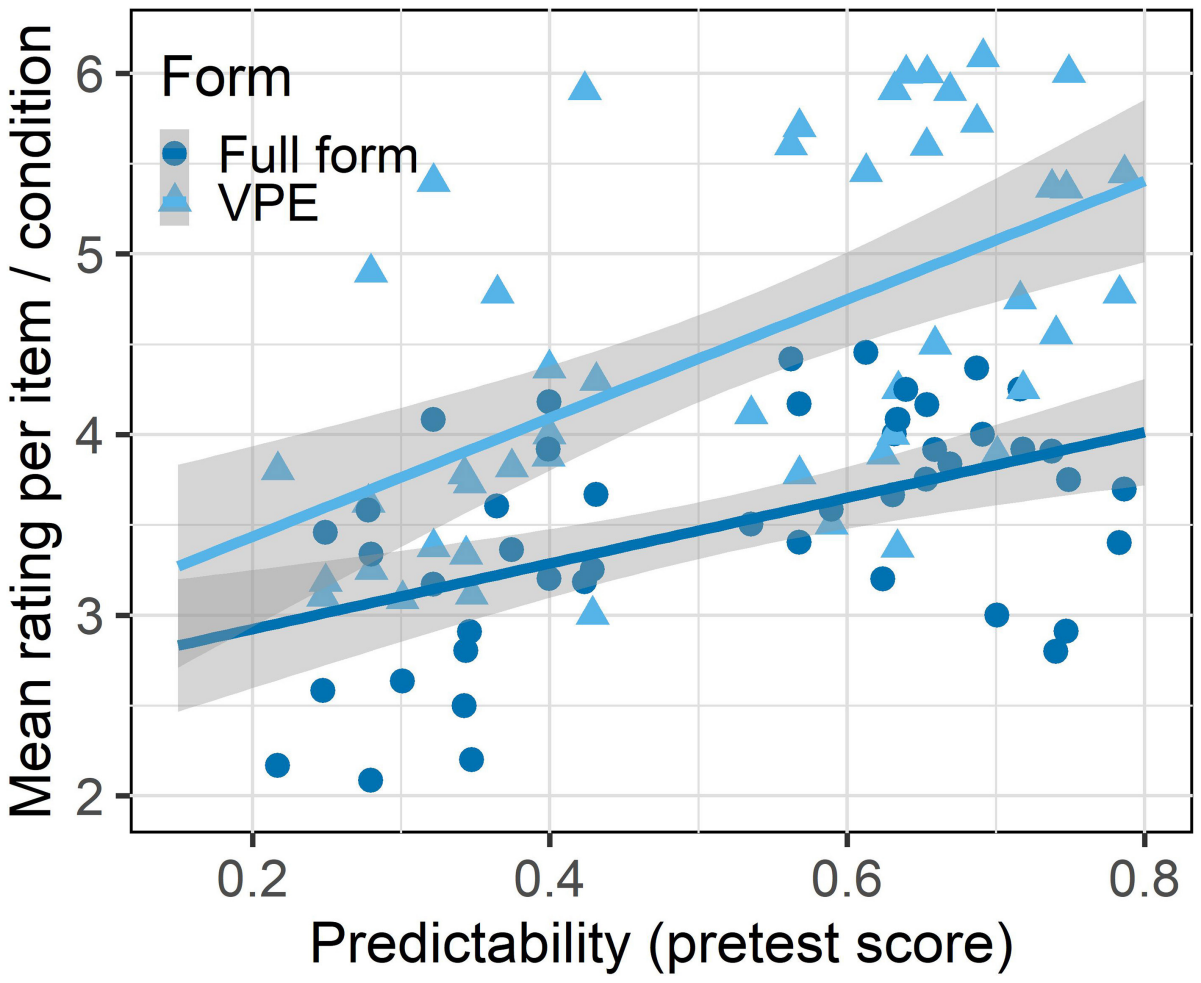
Mean rating per item and condition as a function of the numerical pretest score indicating Predictability for experiment 4.

The final model (Table 3) contains a significant main effect of Form (χ^2^ = 9.75, *p* < 0.01) which indicates a preference for VP ellipses over full forms. We also find a significant main effect of the Predictability score (χ^2^ = 27.58, *p* < 0.001): Utterances that are predictable given the previous context received better ratings. The interaction between Form and Predictability is marginal (χ^2^ = 3.19, *p* = 0.07) and therefore not part of the final model. There is a trend toward better ratings for VP ellipsis in predictive contexts as illustrated in Figure 7.

**Table 3:** Fixed effects in the final clmm for experiment 4.

**Predictor**	**Estimate**	**SE**	**χ^2^**	***p*-value**	
Predictability	4.61	0.76	27.58	< 0.001	^***^
Form	–1.74	0.54	9.75	< 0.01	^**^
Position	0.16	0.13	1.6	> 0.2	
Form:Position	–0.53	0.24	4.89	< 0.05	^*^

* *p < 0.05*,

** *p < 0.01, and*

*** *p < 0.001*.

#### 4.2.4. Discussion

In this rating study, we investigated predictability in context as a source of redundancy for a repeated verb phrase. Our UID account predicts that VP ellipsis should be more strongly preferred when the omitted verb phrase is more predictable in context. In the data, we do not find this predicted interaction between the predictability and the form of the redundant verb phrase. There is only a marginal effect in the expected direction. While the pre-test evidenced a clear difference in likelihood between the two context conditions, this does not result in a stronger preference for VP ellipsis. We find however that our predictability manipulation works: Participants preferred utterances in predictive contexts over such in neutral contexts. Similar to the length rating study, there was also a general preference for the more compact VP ellipsis over the long redundant full forms which is also predicted by the DOAP principle and the repetition penalty account.

So why is there only a marginal preference for VP ellipsis in the predictive conditions? A possible explanation might be that our context manipulation did not affect VP ellipsis because the verb phrase is still too predictable even in our neutral conditions and therefore VP ellipsis is also preferred in these conditions according to UID. Regardless of whether the VP ellipsis follows a predictive or a neutral context, there is always a parallel first verb phrase available which is straightforwardly accessible as antecedent for the ellipsis. Thus, VP ellipsis can be easily processed even in the neutral condition and there is no need to use the redundant full form. This is supported by the overall preference for VP ellipsis over the full form, which we did find in both naturalness rating studies presented in this article.

We further need to consider that the set of possible encodings for the message that Sam played football and that Dean played football does not consist only of the full form and the corresponding VP ellipsis. An alternative encoding is a simple sentence with a coordinated subject like (15) which might be a competitor to the full form but which cannot be readily compared to the other two forms with UID.

(15) Sam and Dean played football in the backyard of the house.

We will turn back to these potential issues in section 5.

### 4.3. Experiment 5 – Self-paced Reading Study

In a 1 × 2 (Context: predictive × neutral) self-paced reading study we investigate whether the context impacts the processing effort on the redundant verb phrase. Our UID based account predicts that the redundant repeated VP is read faster in a predictive compared to a neutral context. This speed-up would evidence deeper regions of low information, i.e., the under-utilization of the hearer's processing resources. For the length effects, we found both degraded ratings and a longer trough for the more redundant full forms. For the context effects, we want to test whether a predictable verb phrase leads to a deeper trough in the information density profile indexed by faster reading times. If we did not find such an effect, i.e., if there was no speed-up in the predictive condition, this would explain why we did not find the expected interaction in the rating study, i.e., why VP ellipsis was not more strongly preferred in the predictive contexts.

#### 4.3.1. Materials

We used the same materials as in experiment 4, but tested only the full forms, both of the items (16) and the fillers. The method is similar to experiment 2, but instead of comparing the reading times between the first and the second verb phrase we compare the reading times on only the second verb phrase between both Context conditions.


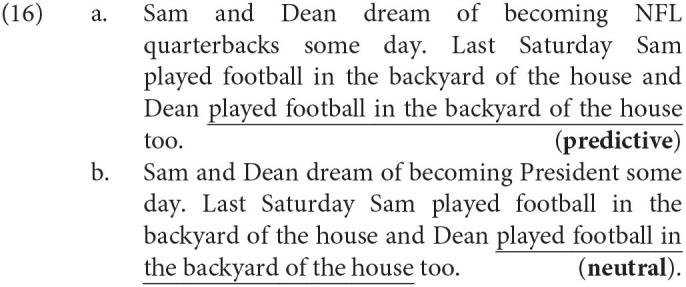


#### 4.3.2. Procedure

49 self-reported native speakers of American English who had not participated in any of the previous experiments were recruited over Prolific Academic to take part in the study.^16^ They received a compensation of £2. We conducted the self-paced reading experiment over the Internet using IBEX. In each trial, subjects first saw the context sentence as a whole and then read the target utterance word-by-word^17^ in a centered self-paced reading paradigm. Before the actual experiments subjects passed a practice phase consisting of 7 sentences and 3 comprehension questions. Materials were distributed across two lists with a Latin square design. In the main experiment each participant read 24 items (12 in each condition) and 84 fillers presented in fully randomized order. Sixteen fillers had a subsequent polar comprehension question that served as attention checks.

In our analysis, we compared the residualized cumulated reading times (RCRT) calculated as described in section 3.2.3 for the identical second VP between the predictive and the neutral condition. We excluded by-word reading times faster than 90 ms and slower than 3,000 ms and all regions of interest that have become incomplete due to these by-word exclusions. This resulted in a loss of about 1% of all regions of interest.

#### 4.3.3. Results

**Figure 8:**
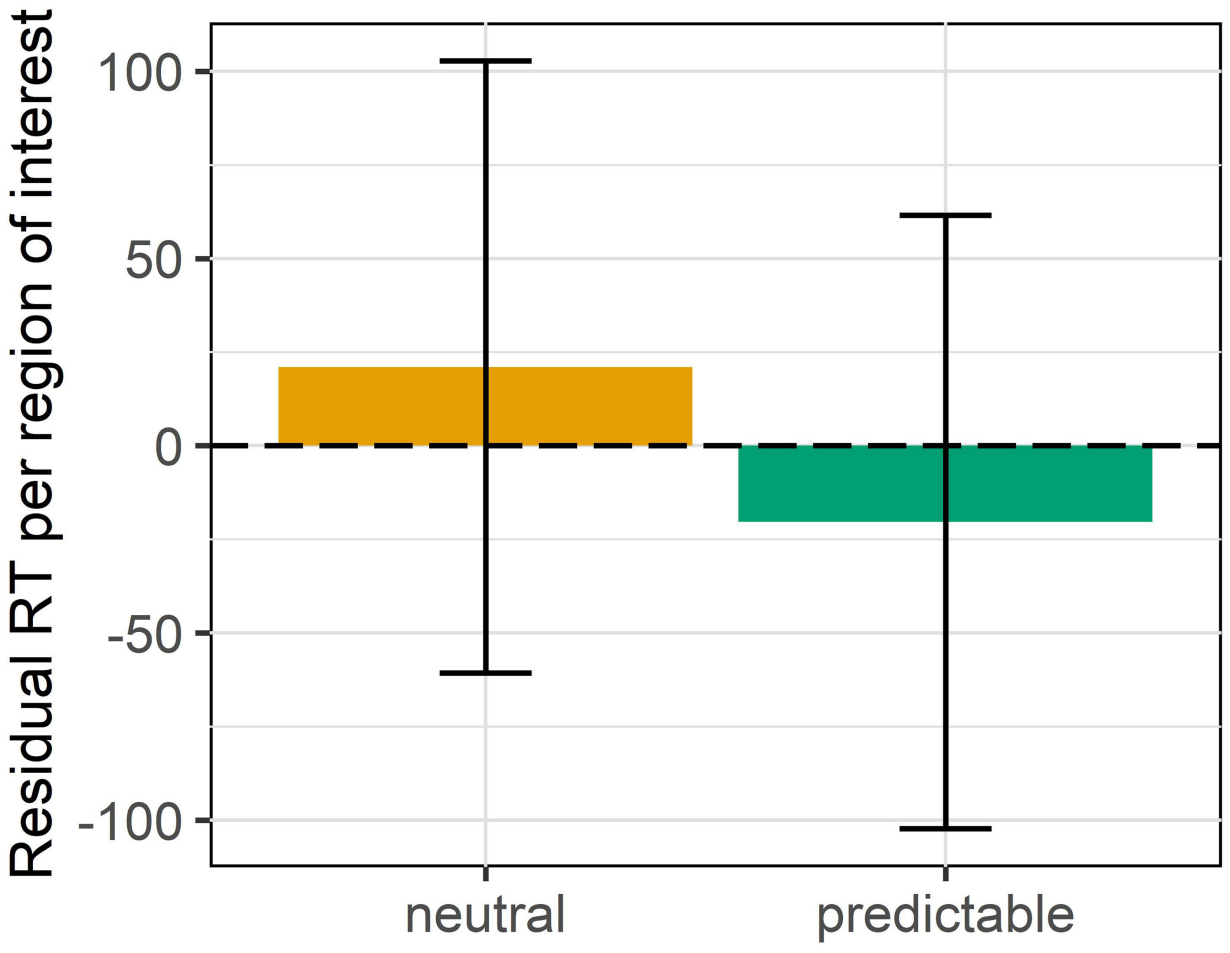
Mean residual reading times and 95% confidence intervals per region of interest per condition for experiment 5.

Before the analysis we excluded 6 participants who had failed our attention checks in having answered more than 4 of 16 comprehension questions incorrectly. We analyzed the data of the remaining 43 participants in R using linear mixed effects models (Bates et al., 2015) and the same procedure of backward model section described in experiment 1 in section 3.1.3. Our full model contained the RCRTs as dependent variable and as independent variables the numerical pre-test score indicating Predictability, the scaled and centered Position of the item in the experiment and their interaction. We only included a random intercept for items because the reading times are already normalized per subject and more complex random effect structures resulted in singular fit.^18^ The final model (Table 4) contained only a significant main effect of Position (χ^2^ = 87.55, *p* < 0.001) indicating that participants became faster in the course of the experiment. The main effect of predictability was not significant (χ^2^ = 0.63, *p* = 0.43). The redundant VP did not differ in reading times between the predictive and the neutral conditions (Figure 8).

**Table 4:** Fixed effects in the final lmer for experiment 5.

**Predictor**	**Estimate**	**SE**	**df**	**χ^2^**	***p*-value**	
Position	–158.91	16.26	486.81	87.55	< 0.001	^***^

^*^p < 0.05,

^**^p < 0.01,

*** *p < 0.001*.

#### 4.3.4. Discussion

We investigated the processing of a redundant verb phrase in a predictive vs. a neutral context and found no difference in reading times of the second redundant verb phrase between context conditions. Specifically, participants did not show a speed-up on the repeated verb phrase after a predictive compared to a neutral context. This way, the results of the self-paced reading study pattern with the results of the rating study in section 4.2. This suggests that the repeated VP is equally redundant in both context conditions. The predictive context does not lead to a deeper information minimum in the information density profile than the neutral context. In section 4.2.4, we already presented a possible explanation for why we do not find the context effects that a UID account would predict. For the self-paced reading study, we add that we presented full forms that are highly unnatural in both conditions given that the second verb phrase is completely identical to the first verb phrase and that a simpler alternative in the form of a sentence with a coordinated subject would be available. This intuition is confirmed by the results of both rating studies in this paper where the long redundant full forms received degraded ratings. We hypothesize that during the reading task this unnaturalness masked the effect of the more subtle context manipulation or even led to severe processing difficulties that resulted in an equally strong slow down for both context conditions.

## 5. General Discussion

We present a novel information-theoretic account to the underexplored question of when VP ellipsis is used. According to the UID hypothesis an increased redundancy leads to information minima which speakers tend to avoid when producing utterances. VP ellipsis or ellipsis in general is a possible strategy to avoid such troughs: The redundant material is omitted or at least drastically reduced. We investigated length and predictability in context as two sources of redundancy of the repeated verb phrase. A longer repeated verb phrase should cause a longer information minimum, while a repeated verb phrase in a predictive compared to a neutral context should result in a deeper information minimum. In both cases, these minima underutilize the hearer's processing resources and we expect that this is reflected in degraded naturalness ratings and faster reading times.

For the length effects manipulation, our results are in line with the predictions of our UID account. In the rating study we found that VP ellipsis is especially preferred over the full form when the redundant verb phrase is longer. In this case also the corresponding information minimum is longer which is equivalent to the underutilizing of the hearer's processing resources for a longer time. In a self-paced reading study we could evidence that the naturalness pattern is caused by processing: The redundant second verb phrase was read relatively faster compared to the first verb phrase when it was longer which indicates a longer information mimimum. The length of the redundant material seems to be indeed a factor that affects the information density profile and hence the usage of VP ellipsis. It is an advantage of our UID account over the DOAP principle (Williams, 1997) and the repetition penalties accounts (e.g., Gordon et al., 1993; Kertz, 2010) that it does not only predict a general categorical penalty for the repetition of redundant material, but a gradual effect of length.

We could not evidence an effect of predictability in context on the redundant verb phrase. In the naturalness rating study we found a non-significant trend toward a preference for VP ellipsis in predictive contexts. In the self-paced reading study, the reading times of the redundant verb phrases did not differ regardless of whether the verb phrase followed a predictive or a neutral context. We identified two possible explanations for this result: (i) The unnaturalness of the long redundant verb phrases could mask more subtle effects. The rating study on length effects evidenced that the long redundant full forms received particularly bad ratings. However, we had to use these full forms in the context studies in order to have enough material to measure on in the self-paced reading study. Since the context manipulation is more subtle than the length manipulation, the effect of the context might be overridden by the penalty caused by the long redundant full form. (ii) It might be the case that our context manipulation itself is too subtle. From a UID perspective there is no need for the speaker to use the full forms in any of the conditions that we tested. The form of our items entails that the first verb phrase is always immediately available as an antecedent for ellipsis. Hence, the ellipsis can be straightforwardly resolved even in the neutral context conditions. VP ellipsis as the shorter form always has an advantage over the less well-formed full form. This, in a future study, it may be promising to find a way to make the VP ellipsis less redundant. That is, the verb phrase should not be highly predictable through a given identical first verb phrase and the discourse connective *and*. A starting point might be to look at cases where the antecedent of the VP ellipsis differs in its morphosyntactic properties from the reconstruction of the ellipsis site. Arregui et al. (2006) tested structures like (17) where the antecedent is not a verb phrase but a gerund or a nominalization. In such cases a UID account could argue that an increased mismatch in form results in decreased redundancy of the repeated verb phrase. A full form as more explicit form could reduce the processing effort here because the effort associated with the more difficult resolving of ellipsis is canceled.


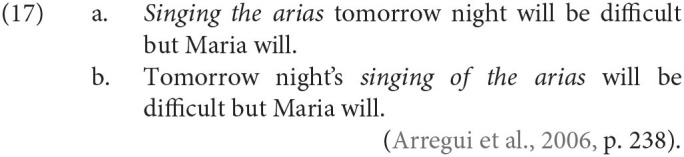


In sum, we find partial support for our information-theoretic account to the usage of VP ellipsis. While the results on length effects are in line with our account based on UID, the results on context effects are not. The context reading study suggests that for structural reasons the redundant verb phrase is still too predictable even in the neutral contexts. This does not provide evidence against UID, but further studies in which VP ellipsis is made less redundant are needed to strengthen our account.

## Data Availability Statement

The raw data supporting the conclusions of this article will be made available by the authors, without undue reservation.

## Ethics Statement

The studies involving human participants were reviewed and approved by the Ethics committee of the Deutsche Gesellschaft für Sprachwissenschaft (German society for language science). The patients/participants provided their written informed consent to participate in this study.

## Author Contributions

LS was responsible for preparing and conducting the experiments, for analyzing and visualizing the resulting data and writing the initial draft of this paper. RL supported LS in preparing and conducting the experiments and in the analysis of the data, and critically commented on the initial draft of this paper. HD and IR developed and formulated the overarching research goals, managed and supervised the research activities, and critically reviewed the analysis of the data and the initial draft of this paper. All authors contributed to the article and approved the submitted version.

## Conflict of Interest

The authors declare that the research was conducted in the absence of any commercial or financial relationships that could be construed as a potential conflict of interest.

## Footnotes

^1^We use the term “hearer” to refer to the recipient of the communication, regardless of whether this communication is auditory or written.

^2^Lemke, R., Schäfer, L., and Reich, L. (under review). *Can identity conditions on ellipsis be explained by processing principles?*

^3^All figures in this paper were created with the package ggplot2 (Wickham, 2016) in R (R Core Team, 2020).

^4^We would like to thank two anonymous reviewers for pointing this out.

^5^The items of all experiments can be found in the Supplementary Materials.

^6^https://www.limesurvey.org/

^7^Ratings ~ (Form + Length + Position)^2 + (1 + (Form + Length + Position)^2 | Subjects) + (1 + (Form + Length + Position)^2 | Items).

^8^We argue that the degraded ratings are caused by redundancy and hence expect a gradual effect, i.e., the ratings get worse the more redundant the target utterance is. A reviewer suggests to test this prediction with partially redundant utterances, such as (i), where the PP is repeated but the core VP is new. (i) a. Sam played football in the backyard of the house and Dean flew a kite in the backyard of the house. b. Sam played football in the backyard of the house and Dean flew a kite there. We must leave a systematic investigation of such cases to future research, but we have tentative data from experiment 4, where a part of our fillers had a similar structure (see section 4.2.1): In this experiment the fully redundant long full forms received a mean rating of 3.82 (σ = 1.92) and the corresponding ellipses got 5.1 (σ = 1.67). With a mean rating of 4.24 (σ = 1.82), the partly redundant full forms with a new second VP and a repeated PP (i-a) lie between these two, which could be a hint toward a gradual effect of redundancy on naturalness ratings. However, this result is questioned by the fact that the corresponding partly reduced forms (i-b) are rated best (5.32, σ = 1.56), even better than the completely reduced forms.

^9^https://spellout.net/ibexfarm/

^10^We adapted the code provided by Gibson and Levy (2016) at https://osf.io/swyux/.

^11^The higher number of exclusions as compared to experiment 1 might be the result of the more demanding reading task. In particular the long repeated VPs might have led to fatigue and consequently to stronger inattention.

^12^RCRT ~(Length + VP + Position)^2 + (0 + VP || Subjects) + (1 + Length | Items).

^13^An example of the latter is given in (i). (i) a. Jodie and Donna were eager to see the new season of their favorite show. Donna watched television on the sofa in the living room. b. Jodie and Donna were eager to go for a jog in the park. Donna watched television on the sofa in the living room.

^14^We thank Elisabeth Rabs for providing us with the original German materials as used in Rabs et al. (under review): *Situational Expectancy or Word Association? The Influence of Event Knowledge on the N400*.

^15^Ratings ~(Form + Predictability + Position)^2 + (1 + Predictability + Position | Subjects) + (1 + (Form + Predictability)^2 + Position | Items).

^16^Due to internal processes of the crowd sourcing platform Prolific, we had the complete data of 49 instead of the planned 48 participants.

^17^An anonymous reviewer suggested that a phrase-by-phrase presentation could help to isolate effects in a clearer way. We will consider this for future studies.

^18^RCRT ~(Predictability + Position)^2 + (1 | Items).
