# Supplementary material for: The Role of UID for the Usage of Verb Phrase Ellipsis: Psycholinguistic Evidence From Length and Context Effects
Source: Front Psychol. 2021 May 26;12:661087. doi: 10.3389/fpsyg.2021.661087 (PMC8189427; doi:10.3389/fpsyg.2021.661087)

frontiers

Supplementary Material

1 ITEMS FOR EXPERIMENTS 1 AND 2 ON LENGTH EFFECTS

Since we tested speakers of British English in experiments and 2, the items were lexicalized accordingly. The placeholders *X*, *Y* and *Z* were automatically replaced with the most frequent first names from the UK, in such a way that no name was used more than once.

(1) X drove cars (in the race on the street) and Y [drove cars (in the race on the street) | did] too *whereas Z preferred to watch*.

(2) X played football (in the backyard of the house) and Y [played football (in the backyard of the house) | did] too *whereas Z studied for university*.

(3) X drank beer (in the pub around the corner) and Y [drank beer (in the pub around the corner) | did] too *while Z ate a burger*.

(4) X watched television (on the sofa in the living room) and Y [watched television (on the sofa in the living room) | did] too *while Z read the newspaper*.

(5) X bought clothes (in the store by the bridge) and Y [bought clothes (in the store by the bridge) | did] too *while Z was waiting in the car*.

(6) X rode horses (at the ranch beyond the pass) and Y [rode horses (at the ranch beyond the pass) | did] too *while Z polished the saddles*.

(7) X fed birds (in the park in the neighbourhood) and Y [fed birds (in the park in the neighbourhood) | did] too *whereas Z played frisbee*.

(8) X ordered coffee (at the cafe behind the office) and Y [ordered coffee (at the cafe behind the office) | did] too *while Z preferred tea*.

(9) X started school (in the county down the road) and Y [started school (in the county down the road) | did] too *while Z stayed in daycare*.

(10) X ate fish (at the restaurant on the pier) and Y [ate fish (at the restaurant on the pier) | did] too *whereas Z just drank a coke*.

(11) X threw confetti (at the celebration in the penthouse) and Y [threw confetti (at the celebration in the penthouse) | did] too *while Z opened the champagne*.

(12) X cooked pasta (in the kitchen on the stove) and Y [cooked pasta (in the kitchen on the stove) | did] too *while Z ordered pizza*.

(13) X booked holidays (in the travel agency at the mall) and Y [booked holidays (in the travel agency at the mall) | did] too *whereas Z is staying at home this year*.

(14) X waved flags (in the parade at the square) and Y [waved flags (in the parade at the square) | did] too *while Z played in the band*.

(15) X trained dogs (in the yard behind the shelter) and Y [trained dogs (in the yard behind the shelter) | did] too *while Z went for a walk*.

(16) X shared information (at the workplace in the meeting) and Y [shared information (at the workplace in the meeting) | did] too *while Z finished the report*.

(17) X studied medicine (at the university in Denver) and Y [studied medicine (at the university in Denver) | did] too *whereas Z became an actress*.

(18) X denied involvement (in the inspectors office at the station) and Y [denied involvement (in the inspectors office at the station) | did] too *while Z confessed the crime*.

(19) X met friends (in the plaza near the apartment) and Y [met friends (in the plaza near the apartment) | did] too *while Z worked on Zpos thesis*.

(20) X skipped stones (in the playground by the lake) and Y [skipped stones (in the playground by the lake) | did] too *while Z was playing tag*.

(21) X collected firewood (in the forest behind the cabin) and Y [collected firewood (in the forest behind the cabin) | did] too *whereas Z pitched the tent*.

(22) X corrected papers (in the teacher's lounge at the school) and Y [corrected papers (in the teacher's lounge at the school) | did] too *whereas Z prepared the next lesson*.

(23) X wrapped gifts (in the living room at the desk) and Y [wrapped gifts (in the living room at the desk) | did] too *while Z decorated the tree*.

(24) X gathered mushrooms (in the woods near the church) and Y [gathered mushrooms (in the woods near the church) | did] too *while Z was flying a kite*.

(25) X climbed mountains (in the countryside near the river) and Y [climbed mountains (in the countryside near the river) | did] too *whereas Z was afraid of heights*.

(26) X browsed websites (at the hotel in the lobby) and Y [browsed websites (at the hotel in the lobby) | did] too *whereas Z was taking a shower*.

(27) X sold antiques (at the flea market near the square) and Y [sold antiques (at the flea market near the square) | did] too *while Z gave away furniture*.

(28) X chaired meetings (at the building behind the garage) and Y [chaired meetings (at the building behind the garage) | did] too *while Z took the minutes*.

(29) X produced music (in the studio in the basement) and Y [produced music (in the studio in the basement) | did] too *while Z adjusted the microphones*.

(30) X practised yoga (at the gym by the market) and Y [practised yoga (at the gym by the market) | did] too *while Z was lifting weights*.

(31) X brought wine (to the gathering in the park) and Y [brought wine (to the gathering in the park) | did] too *whereas Z came empty-handed*.

(32) X celebrated success (at the party by the pool) and Y [celebrated success (at the party by the pool) | did] too *while Z prepared a presentation*.

2 ITEMS FOR EXPERIMENTS 4 AND 5 ON CONTEXT EFFECTS

In experiments 4 and 5 we tested speakers of American English, so the items were slightly changed as compared to experiments 1 and 2. The placeholders *X*, *Y* and *Z* were automatically replaced with the most frequent first names from the U.S., in such a way that no name was used more than once.

(1) a. X and Y like motor sports. (**predictive**)
b. X and Y like sports. (**neutral**)
c. Last Saturday X drove cars in the race on the street and Y [drove cars in the race on the street | did] too.

(2) a. X and Y dream of becoming NFL quarterbacks some day. (**predictive**)
b. X and Y dream of becoming President some day. (**neutral**)
c. Wednesday afternoon X played football in the backyard of the house and Y [played football in the backyard of the house | did] too.

(3) a. X and Y enjoy going out on the weekends. (**predictive**)
b. X and Y enjoy mountain biking in the forests. (**neutral**)
c. Last Friday X drank beer in the pub around the corner and Y [drank beer in the pub around the corner | did] too.

(4) a. X and Y were eager to see the new season of their favorite show. (**predictive**)
b. X and Y were eager to go for a jog in the park. (**neutral**)
c. On Saturday X watched television on the sofa in the living room and Y [watched television on the sofa in the living room | did] too.

(5) a. X and Y needed to update their wardrobes. (**predictive**)
b. X and Y needed to get groceries. (**neutral**)
c. After work X bought clothes in the store by the bridge and Y [bought clothes in the store by the bridge | did] too.

(6) a. X and Y had been looking forward to their cowboy excursion. (**predictive**)
b. X and Y had been looking forward to their trip. (**neutral**)
c. Last week X rode horses at the ranch beyond the pass and Y [rode horses at the ranch beyond the pass | did] too.

(7) a. X and Y enjoy spending time at the pond in the afternoons. (**predictive**)
b. X and Y enjoy spending time at the movies in the afternoons. (**neutral**)
c. After lunch X fed birds in the park in the neighbourhood and Y [fed birds in the park in the neighbourhood | did] too.

(8) a. X and Y had to get an energy boost before work. (**predictive**)

b. X and Y had to make a call before work. (**neutral**)
c. At 7AM X ordered coffee at the cafe behind the office and Y [ordered coffee at the cafe behind the office | did] too.

(9) a. X and Y were excited to start learning how to write. (**predictive**)
b. X and Y were excited to start learning how to ride a bike. (**neutral**)
c. Last month X started school in the county down the road and Y [started school in the county down the road | did] too.

(10) a. X and Y wanted to get seafood for dinner. (**predictive**)
b. X and Y wanted to get a nice meal for dinner. (**neutral**)
c. Yesterday evening X ate fish at the restaurant next to the station and Y [ate fish at the restaurant next to the station | did] too.

(11) a. X and Y like to eat Italian food. (**predictive**)
b. X and Y like dining out. (**neutral**)
c. On Tuesday X cooked pasta in the kitchen on the stove and Y [cooked pasta in the kitchen on the stove | did] too.

(12) a. X and Y had their requests for time off approved. (**predictive**)
b. X and Y had their meeting with their bosses. (**neutral**)
c. After work X booked holidays in the travel agency at the mall and Y [booked holidays in the travel agency at the mall | did] too.

(13) a. X and Y love patriotic holidays. (**predictive**)
b. X and Y love huge events. (**neutral**)
c. Last weekend X waved flags in the parade at the square and Y [waved flags in the parade at the square | did] too.

(14) a. X and Y like to work in teams. (**predictive**)
b. X and Y like to work alone. (**neutral**)
c. This morning X shared information at the workplace in the meeting and Y [shared information at the workplace in the meeting | did] too.

(15) a. X and Y had planned the heist together. (**predictive**)
b. X and Y had planned the trip together. (**neutral**)
c. Two weeks later X denied involvement in the inspectors office at the station and Y [denied involvement in the inspectors office at the station | did] too.

(16) a. X and Y were feeling social yesterday. (**predictive**)
b. X and Y were very busy yesterday. (**neutral**)
c. At 5PM X met friends in the plaza near the center and Y [met friends in the plaza near the center | did] too.

(17) a. X and Y like to spend time near the waterfront. (**predictive**)
b. X and Y like to spend time at home. (**neutral**)
c. After school X skipped stones in the playground by the lake and Y [skipped stones in the playground by the lake | did] too.

(18) a. X and Y go camping frequently. (**predictive**)
b. X and Y frequently go swimming at the lake. (**neutral**)
c. This evening X collected firewood in the forest behind the cabin and Y [collected firewood in the forest behind the cabin | did] too.

(19) a. X and Y are always excited for Christmastime. (**predictive**)
b. X and Y are always excited for family gatherings. (**neutral**)
c. On Thursday X wrapped gifts in the living room at the table and Y [wrapped gifts in the living room at the table | did] too.

(20) a. X and Y like to go foraging for ingredients outdoors. (**predictive**)
b. X and Y like to go running in the park outdoors. (**neutral**)
c. In the morning X gathered mushrooms in the woods near the church and Y [gathered mushrooms in the woods near the church | did] too.

(21) a. X and Y have trouble concentrating at work. (**predictive**)
b. X and Y work at an insurance company. (**neutral**)
c. This afternoon X browsed websites at the computer in the office and Y [browsed websites at the computer in the office | did] too.

(22) a. X and Y gathered their recording equipment yesterday. (**predictive**)
b. X and Y gathered their professional tools yesterday. (**neutral**)
c. In the morning X produced music in the studio in the basement and Y [produced music in the studio in the basement | did] too.

(23) a. X and Y hate to show up without bringing a gift. (**predictive**)
b. X and Y hate to arrive later than everyone else. (**neutral**)
c. Last Saturday X brought wine to the gathering in the park and Y [brought wine to the gathering in the park | did] too.

(24) a. X and Y like to pat themselves on the back after closing a deal. (**predictive**)
b. X and Y like to buckle down and work hard after closing a deal. (**neutral**)
c. Last weekend X celebrated success at the party by the pool and Y [celebrated success at the party by the pool | did] too.

3 WORD-BY-WORD READING TIME PLOTS FOR EXPERIMENTS 2 AND 5

We show word-by-word reading time plots for experiment 2 (left) and experiment 5 (right). We plot the mean raw reading times per word type and condition plus 95% confidence intervals for the complete critical sentence including spillover region as exemplified in (1). Note that the PPs in the long conditions vary in length and that we consequently have fewer data points for PP1_6, PP1_7, PP2_6 and PP2_7 resulting in larger confidence intervals.

(1) (Last Saturday) Sam played football in the backyard of the house and
(Initial Adv) NAME1 V1 O1 PP1_1 PP1_2 PP1_3 PP1_4 PP1_5 PP1_6 AND
Dean played football in the backyard of the house too whereas Jack
NAME2 V2 O2 PP2_1 PP2_2 PP2_3 PP2_4 PP2_5 PP2_6 TOO S1 S2
studied for university.
S3 S4 S5

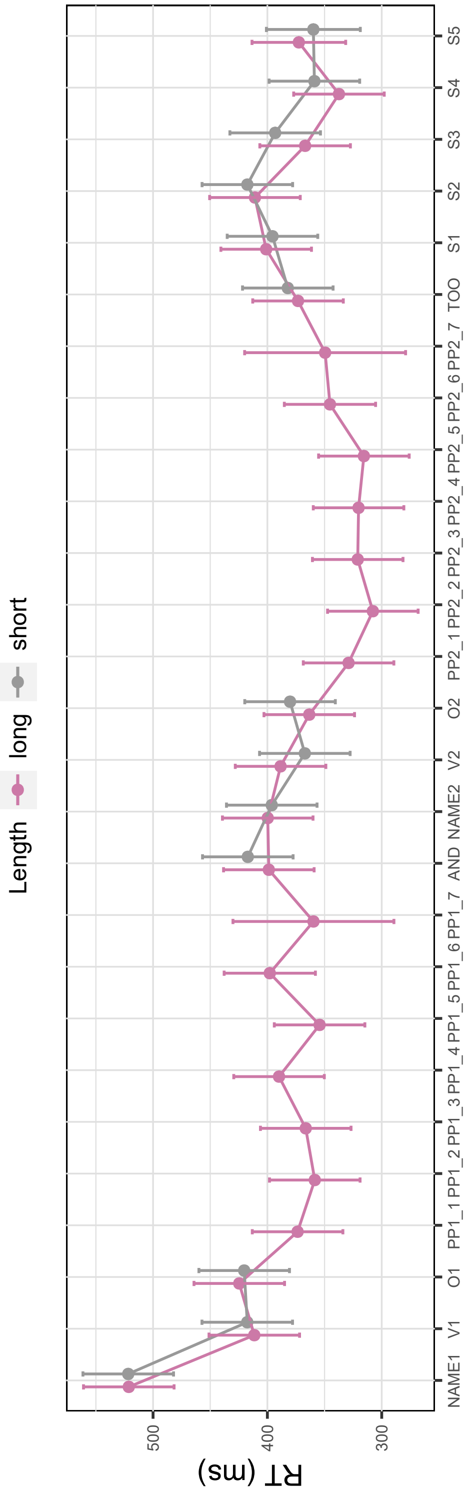

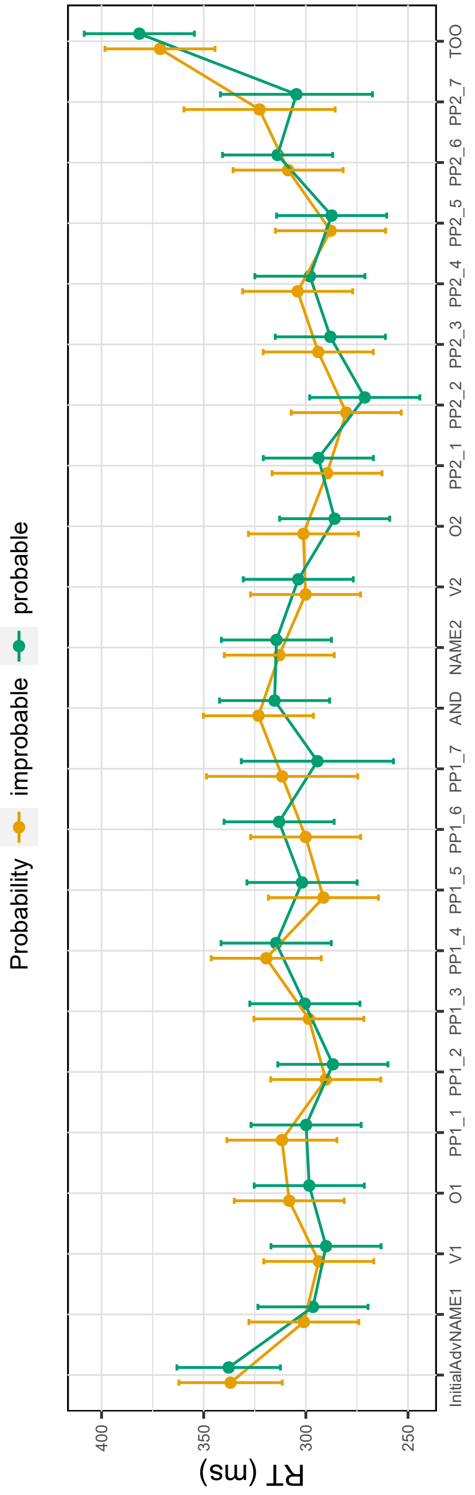

Supplement: Supplementary file 1 [file Data_Sheet_1.pdf]
